# Initiating discussions about weight in a non‐weight‐specific setting: What can we learn about the interactional consequences of different communication practices from an examination of clinical consultations?

**DOI:** 10.1111/bjhp.12322

**Published:** 2018-06-26

**Authors:** Susan A. Speer, Rebecca McPhillips

**Affiliations:** ^1^ Division of Psychology and Mental Health School of Health Sciences The University of Manchester UK

**Keywords:** clinical communication, conversation analysis, medical interaction, obesity, overweight, psychiatry, weight

## Abstract

**Objectives:**

Effective clinical communication is fundamental to tackling overweight and obesity. However, little is known about how weight is discussed in non‐weight‐specific settings where the primary purpose of the interaction concerns clinical matters apparently unrelated to weight. This study explores how mental health clinicians initiate discussions about a patient's possible weight problem in the non‐weight‐specific setting of a UK NHS Gender Identity Clinic (GIC), where weight is topicalized during discussions about the risks of treatment.

**Design:**

A conversation analytic study.

**Methods:**

A total of 194 recordings of routine clinician–patient consultations were collected from the GIC. Weight talk was initiated by four clinicians in 43 consultations. Twenty‐one instances contained reference to a possible weight problem. Transcripts were analysed using conversation analysis.

**Results:**

Clinicians used three communication practices to initiate discussion of a possible weight problem with patients: (1) *announcing* that patients are overweight; (2) *asking* patients whether they are overweight; and (3) *deducing* that patients are overweight or obese via a body mass index (BMI) calculation. *Announcing* that patients are overweight is the least aligning practice that denies patient's agency and grammatically constrains them to agree with a negative label. *Asking* patients whether they are overweight treats them as having limited agency and generates comparatively aligning, but occasionally resistant, responses. Jointly *deducing* that patients are overweight or obese via a BMI calculation is the most aligning practice, which deflects responsibility for labelling the patient onto an objective instrument.

**Conclusions:**

Small differences in the wording of turns that initiate discussions about a possible weight problem can have significant consequences for interactional alignment. Clinicians from different specialities may benefit from considering the interactional consequences of different practices for initiating discussions about weight during the kinds of real‐life discussions considered here.

**Statement of contribution**

***What is already known on this subject?***

- There is a correlation between clinical communication about weight and patient weight loss.
- Clinicians from all specialties are encouraged to discuss diet and exercise with patients, but communication about weight remains problematic.
- Health psychologists have identified an urgent need for communication training to raise sensitive topics like weight without damaging the doctor–patient relationship.

***What does this study add?***

- Clinicians in a non‐weight‐specific setting use three communication practices to introduce the possibility that a patient's weight may be a problem.
- These practices have varying consequences for the interaction and doctor–patient relationship.
- Conversation analytic findings may be useful in training clinicians how to initiate discussions about weight with patients.

## Background

In 2016, it was estimated that 39 per cent of adults were overweight and 13 per cent were obese, signalling a global health problem (World Health Organisation, 2018). Research shows a positive relationship between clinicians addressing patients’ weight and patient weight loss (Huang, Yu, Marin, Brock, & Davis, 2004; Kant & Miner, 2007; Loureiro & Nayga, 2006; Pollak *et al*., 2010; Rose, Poynter, Anderson, Noar, & Conigliaro, 2013). However, discussing weight with patients remains problematic (Dewhurst, Peters, Devereux‐Fitzgerald, & Hart, 2017; Finset, 2009; Kushner, 2011). Clinicians report that they lack knowledge regarding measures including body mass index (BMI), struggle to set goals for patients (Blackburn, Stathi, Keogh, & Eccleston, 2015; Dixon, Hayden, O'Brien, & Piterman, 2008; Huang *et al*., 2004; Jay *et al*., 2008), and are apprehensive about addressing weight due to potentially negative reactions (Chisholm, Hart, Lam, & Peters, 2012; Heintze *et al*., 2010; Michie, 2007). Patients expect clinicians to initiate talk about weight (Epstein & Ogden, 2005; Hart, Yelland, Mallinson, Hussain, & Peters, 2016), but report that they appear ambivalent towards weight (Brown, Thompson, Tod, & Jones, 2006; Forhan, Risdon, & Solomon, 2013; Mold & Forbes, 2011), lack knowledge and training (Forhan *et al*., 2013; Mold & Forbes, 2011), and sometimes feel ‘humiliated’ by such discussions (Malterud & Ulriksen, 2010; Mold & Forbes, 2011).

In the United Kingdom, weight management interventions have typically been considered the responsibility of primary care and community settings (Booth, Prevost, & Gulliford, 2015; Laws, 2004; National Institute for Health & Care Excellence, 2012). However, the National Health Service (NHS) Future Forum has recommended that clinicians ‘make every contact count’ by maximizing opportunities to discuss patients’ lifestyle, including diet and exercise, ‘whatever their specialty or the purpose of the contact’ (National Health Service Future Forum, 2011: 10–11).

A collaborative, ‘person‐centred’ communication style that emphasizes shared decision‐making is considered key to this endeavour (Kushner, 2011; NHS RightCare, 2017; NICE, 2006; Strategies to Overcome and Prevent Obesity Alliance (STOP), 2014; Swift, Choi, Puhl, & Glazebrook, 2013). The UK's National Institute for Health and Care Excellence (NICE) recommends that health professionals are trained in ‘the appropriate language to use’ (2012: 33) when discussing weight with patients and that adults are given individually tailored, ‘jargon‐free’ information about their weight and associated health risks (2014: 18). Other guidance includes using the ‘5As’ model, adapted for use in weight counselling (Vallis, Piccinini‐Vallis, Sharma, & Freedhoff, 2013). During the first step, ‘Ask’, clinicians seek permission from patients to talk about weight (e.g., ‘would it be alright if we discussed your weight?’) ( Kushner, 2011; STOP, 2014: 7; Vallis *et al*., 2013: 28). This proactive, ‘interactive’ approach to discussing weight (Kushner, 2011; Scott *et al*., 2004) is supported by research, which shows that patients prefer doctors to ask them whether they want to discuss weight, ‘before they just jump in’ (Ward, Gray, & Paranjape, 2009: 581). Other advice includes avoiding colloquial terms, such as ‘fat’ (Kushner, 2011; Vallis *et al*., 2013), and using ‘non‐judgemental’, ‘people‐first’ language that refers to patients as ‘having obesity’ rather than being ‘obese’ (Strategies to Overcome and Prevent Obesity Alliance, 2014: 5). Research suggests that ‘medicalizing’ weight (e.g., presenting it as a problem in the context of associated conditions, like diabetes) is the least stigmatizing way of addressing it (Schauer, Woodruff, Holz, & Kegler, 2014; Scott *et al*., 2004), and using language that emphasizes achieving or maintaining a ‘healthy weight’ (rather than ‘preventing obesity’) may be more acceptable for some communities (NICE, 2012: 33). Finally, *opportunistic* strategies which use a trigger to transition to talk about health behaviour (such as talk about a health condition like diabetes) lead to greater rates of advice giving in primary care than *structured* strategies, which use a routine such as a new patient form (Flocke, Kelly, & Highland, 2009).

Although this literature is useful for clinicians who wish to discuss weight with patients, it frequently uses ‘expert opinion’ to provide hypothetical examples of communication practices that are assumed to work (Kushner, 2011; Strategies to Overcome and Prevent Obesity Alliance, 2014; Vallis *et al*., 2013; ), *post‐hoc* interviews with clinicians and patients which rely upon memories of interaction that do not always accurately represent what happened (Schauer *et al*., 2014; Ward *et al*., 2009), or observations and recordings of communication which are considered through ‘codes that lock aspects of interaction into a set of predefined strategies’ (Maynard & Heritage, 2005: 428; Flocke *et al*., 2009; Heintze *et al*., 2010; Pollak *et al*., 2007; Scott *et al*., 2004). The disadvantage of these methods is that they neglect the contextualized details of real‐life interactions, which are often ‘messier’ and more nuanced than idealized, invented, or recollected examples.

A small number of studies have used conversation analysis (CA) to examine how weight is discussed in real‐life settings (Pillet‐Shore, 2006; Webb, 2009, 2013, 2015; Wiggins, 2009). However, these studies are based predominantly on data from weight‐specific settings (e.g., a weight management clinic), where it is expected that weight will be discussed. Despite calls to make ‘every contact count’ (NHS Future Forum, 2011), little is known about how weight is addressed in non‐weight‐specific settings where the primary purpose of the interaction is to discuss matters that are apparently unrelated to weight, and where raising the topic of weight is an arguably more complex task.

Our aim in this study is to identify the communication practices used by clinicians to initiate discussion of a patient's possible weight problem in the non‐weight‐specific setting of a Gender Identity Clinic (GIC). The GIC sees a wide range of individuals with gender identity concerns, including gender dysphoria (GD). GD refers to the ‘discomfort or distress that is caused by a discrepancy between a person's gender identity and that person's sex assigned at birth (and the associated gender role and/or primary and secondary sex characteristics)’ (American Psychiatric Association, 2013; World Professional Association for Transgender Health [WPATH], 2011: 5). Hormone therapy and chest or genital surgery are common treatments for GD (Royal College of Psychiatrists, 2013). In this setting, clinicians sometimes raise the topic of weight: A healthy BMI is important for patients who wish to access medical treatment, as being overweight or obese can exacerbate side effects associated with hormone therapy and pose a risk for surgery (WPATH, 2011). Throughout this study, we use the terms ‘overweight’ and ‘obese’ to reflect their different medical uses (e.g., in BMI measurement categories), while recognizing their different associations with ill health and vulnerability and representations in everyday discourse (Moffat, 2010; Rich & Evans, 2005). In the headings applied to the practices we identify, and when quoting our data, we use the terms of the participants.

## Method

A total of 194 routine clinical consultations were recorded as part of a study conducted at a UK NHS GIC, between 2004 and 2008. In phase 1 of the study, conducted between 2005 and 2006, 156 consultations were successfully audio‐recorded by four psychiatrists. Due to staff changes and varying shift patterns, two male psychiatrists recorded the majority of sessions (1M *n* = 63 consultations, 2M *n* = 75, 3M *n* = 14, 1F *n* = 4). In an amendment to the study, the lead psychiatrist (1M) video‐recorded an additional 38 consultations (1M total *n* = 101).

The sample comprised recordings of 182 consecutive consenting patients at different stages of the assessment and treatment process, from initial intake assessments to exit sessions and post‐surgery follow‐ups. Twelve of the recorded consultations were repeat visits. Consultations lasted between 20 and 99 min (total mean = 41 min; 1M mean = 37 min; 2M = 43 min; 3M = 50 min; 1F = 63 min).

Participant information sheets and consent forms were sent by administrators to patients with their routine appointment letter. Clinicians obtained informed consent from patients and operated the recording devices. Ethical approval was granted by the NHS Central Office of Research Ethics Committees.

Consultations were transcribed verbatim, and the corpus was systematically searched, identifying 43 instances where three of the four participating clinicians initiated discussion about weight with patients. Twenty‐one of these instances involved discussion about a patient's possible weight problem (1M *n* = 12, M2 *n* = 8, F1 *n* = 1; total *n* = 21). Given their frequency and theoretical interest, these 21 instances formed the focus for this study. Of the remaining instances, 11 involved discussions about the relationship between hormone therapy and weight gain, six involved reference to the possibility that any weight gain may be a problem for surgery, two referenced the relationship between polycystic ovarian syndrome and weight gain, two referenced discussion of weight in previous consultations, and one, the patient's historical weight problem.

The 21 instances were transcribed in detail using CA conventions, which represent talk in greater detail than verbatim transcription, so that its subtle nuances are captured and can be analysed (Jefferson, 2004; see Table 1). Recurrent practices of interaction that took place within the 21 instances were identified using CA (Schegloff, 2007; Sidnell, 2013). CA has been used to great effect to identify interactional patterns in clinical settings and to inform clinical practice (Antaki, 2011; Heritage & Maynard, 2006; Maynard & Heritage, 2005; Pilnick, Hindmarsh, & Gill, 2009).

**Table 1:** Transcription symbols (Adapted from Jefferson, 2004)

Aspects of the relative placement/timing of utterances		
=	Equals sign	Immediate latching of successive talk
(0.8)	Time in parentheses	The length of a silence, in tenths of a second
(.)	Period in parentheses	A silence that is less than a tenth of a second
[overlap]	Square brackets	Mark the onset and end of overlapping talk

Aspects of speech delivery		
.	Period	Closing, usually falling intonation
,	Comma	Continuing, slightly upward intonation
?	Question mark	Rising intonation
Underline	Underlining	Talk that is emphasized by the speaker
Rea::lly	Colon(s)	Elongation or stretch of the prior sound
c:	Underlining preceding colon	When letters preceding colons are underlined the pitch rises on the letter and the overall contour is ‘up‐to‐down’
:	Underlined colon	Rising pitch on the colon in an overall ‘down‐to‐up’ contour
!	Exclamation mark	Animated tone
‐	Hyphen/dash	A sharp cut‐off of the prior word or sound
↑	Upward arrow	Precedes a marked rise in pitch
↓	Downward arrow	Precedes a marked fall in pitch
<	‘Greater than’ sign	Talk that is ‘jump‐started’
>faster<	‘Lesser than’ & ‘greater than’ signs	Enclose speeded up or compressed talk
<slower>	‘Greater than’ & ‘lesser than’ signs	Enclose slower or elongated talk
LOUD	Upper case	Talk that is noticeably louder than that surrounding it
°quiet °	Degree signs	Enclose talk that is noticeably quieter than that surrounding it
huh/hah/heh/hih/hoh		Various types of laughter token
(h)	‘h’ in parentheses	Audible aspirations within speech (e.g., laughter particles)
.hhh	A dot before an h or series of hs	An inbreath (number of hs indicates length)
hhh	An h or series of hs	An outbreath/breathiness (number of hs indicates length)
$ or £	Dollar or pound sign	Smile voice
*	Asterisk	Squeaky vocal delivery
( )	Empty single parentheses	Non‐transcribable segment of talk.
(talk)	Word(s) in single parentheses	Transcriber's possible hearing
(it)/(at)	A slash separating word(s) in single parentheses	Two alternative transcriber hearings
((laughs))	Word(s) in double parentheses	Transcriber comments or description of a sound

Detailed analyses proceeded as follows: Taking each instance in turn, transcripts were read alongside the original sound or video file with a view to identifying the main actions or ‘practices’ that were involved in raising the possibility that the patient's weight may be a problem (e.g., ‘announcing’, ‘asking’, ‘deducing’). Instances were then analysed in greater detail by considering the words, phrases and grammatical composition of those practices, and their relative position in the sequence (i.e., we considered what came before the clinician's mention of weight, and how patients responded). Next, we identified examples of the three practices for inclusion in the paper, selecting extracts to show both commonalities and important variations within each practice. Identifying details have been replaced with pseudonyms. Patients are referred to using pronouns that reflect the gender they identify with. Clinicians are identified in extract headers by number and sex (e.g., 1M).

## Results

Analyses are divided into three sections that reflect the communication practices that clinicians used to initiate discussions about a potential weight problem:

1. Announcing that patients are overweight (1M *n* = 9; 1F *n* = 1; total *n* = 10);
2. Asking patients whether they are overweight (1M *n* = 5; 2M *n* = 1; total *n* = 6); and
3. Deducing that patients are overweight or obese by calculating their body mass index (BMI) (1M *n* = 1; 2M *n* = 7; total *n* = 8).

Twenty‐four relevant actions were identified across the 21 instances: Although each clinician appeared to favour a particular communication practice (e.g., 1M favoured announcing, 2M favoured BMI calculations), sometimes they combined practices (e.g., asking and deducing [2M, extract 2d], or announcing, asking, and announcing again [1M]).

### Announcing that patients are overweight

The most commonly used practice for initiating discussion about a possible weight problem involved *announcing* that the patient is overweight (Terasaki, 2004), as shown in extract 1a.


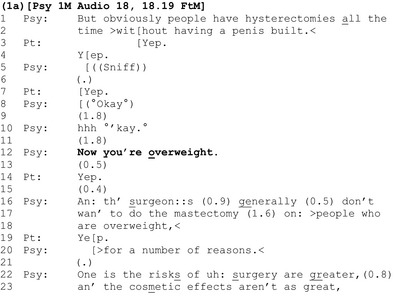


The clinician and patient have been discussing surgical procedures (lines 1–2). Once this discussion comes to completion (line 10), the clinician announces that the patient is ‘overweight’ (line 12).

Introducing weight in this way is interesting: The clinician positions himself as having epistemic access to, and primacy over, the assessable – the patient's size is visually apparent and he produces the ‘overweight’ assessment as an objective fact. Conversation analysts have shown that the interactionally preferred response to a *positive* assessment (e.g., ‘it's a lovely day’) is an immediate and upgraded agreement (e.g., ‘yes, it's beautiful’) (Pomerantz, 1984). However, as a *negative* assessment, the doctor's announcement that the patient is overweight amounts to a criticism of the patient. To agree with a negative assessment of oneself is not straightforward and places conflicting interactional demands on the patient: By agreeing with the announcement, the patient avoids having to challenge the clinician's perception and authority and may ensure that the interaction progresses smoothly. However, an agreement also involves accepting a failure to meet a criteria for the surgery upon which the patient's future depends.

Evidence for disalignment between clinician and patient is apparent in the patient's response: His agreement (‘yep’, line 14) is significantly delayed (see the 0.5‐s gap at line 13), clipped, and hence ‘designedly’ minimal. Rather than seeking to address this apparent disalignment (e.g., by acknowledging that the ‘overweight’ assessment might be difficult to hear), the clinician moves on, apparently masking the interactional turbulence that the announcement generated, by building his next turn as incrementally continuous with it (using the ‘and’ preface) (line 16), and accounting for the relevance of weight to surgery (lines 16ff).

Another clinician tells the patient she is overweight in a similar way in extract 1b, but the patient's response is managed differently.


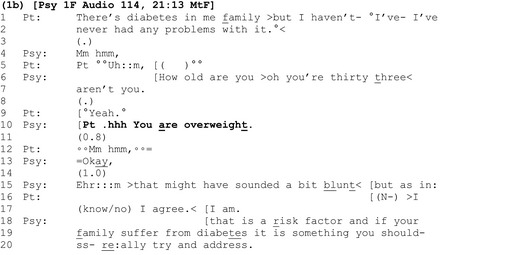


Here again, the announcement ‘you are overweight’ (line 10) follows other health‐related talk (lines 1–2) and the patient's response is both significantly delayed (line 11) and minimal, consisting of a barely audible ‘Mm hmm’ (line 12). However, instead of masking the apparent turbulence and disalignment generated by the assessment like the clinician in extract 1a, here the psychiatrist addresses it head‐on: first by pursuing a response from the patient (line 13), which she does not get (line 14), and then by providing a self‐critical metacommentary on the bluntness of her assessment (line 15 – ‘that might have sounded a bit blunt’).

It is common for the recipient of a self‐deprecation to disagree (Pomerantz, 1984), as happens here: The patient dismisses the suggestion that the assessment sounded blunt and unequivocally agrees with its content (lines 16–17), this time asserting a greater degree of epistemic authority than in her initial response with ‘I agree. I am’ (line 17) (Heritage & Raymond, 2012). Finally, here again, the clinician provides an account which justifies the relevance of her original negative assessment (‘that is a risk factor’) (line 18).

In extracts 1a and 1b, clinicians left a slot for patients to respond following announcements about their weight, before detailing the risks associated with excess weight. By contrast, in extract 1c, the clinician does not wait for the patient to respond before adding a latched question concerning what the patient is going to do about her weight (lines 8–9).


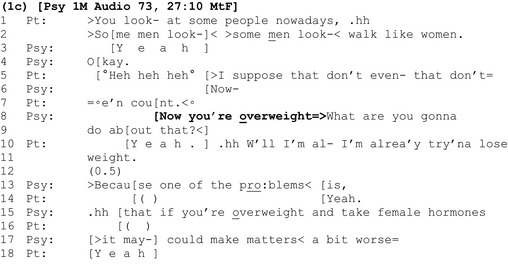


The announcement ‘Now you're overweight’ is delivered following a discussion about procedures that the patient may undergo to live as a woman, including her reflections on walking like a woman (lines 1–2, 5 and 7). The clinician latches a ‘wh’ question immediately after his announcement (lines 8–9). ‘Wh’ questions begin with words such as ‘who’, ‘where’, and ‘what’ and specify the *kind* of answer made relevant by them (Heritage, 2010). Here, the question ‘What are you gonna do about that’ makes the formulation of a future action specific to weight loss, relevant, and presumptively assumes alignment with the negative assessment of the patient's weight *before* securing it.

As before, the patient's agreement with the assessment (‘yeah’, line 10) is delayed, coming at the end of the clinician's latched question. Importantly, the patient undercuts the presupposition in the question that she is *yet* to address her weight by clarifying that this is something she is already working on (lines 10–11) (Heritage, 2010; Schegloff & Lerner, 2009). Clearly, there is disalignment between doctor and patient here. However, instead of addressing this, and the inaccurate presumption highlighted by the patient, the clinician prefaces his next turn about risks with ‘because’, building it as continuous with his prior talk (line 13). As in extract 1a, he masks, and avoids addressing, the interactional turbulence generated by his presupposition.

We have considered three instances where clinicians tell patients they are overweight via an announcement containing a negative assessment. In each case, these announcements generate varying degrees of disalignment between clinician and patient. However, it is only in extract 1b that the clinician directly addresses this disalignment and secures a more aligning response from the patient, before discussing associated health risks.

### Asking patients whether they are overweight

A second way in which clinicians initiate discussions of weight is with a ‘yes/no interrogative’ where patients are asked directly whether they are overweight (Heritage, 2010; Heritage & Raymond, 2012), as shown in extract 2a.


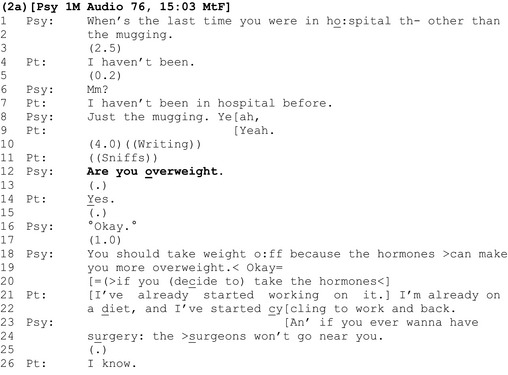


Like the announcements in the previous section, the interrogative ‘are you overweight?’ follows talk about the patient's health. However, unlike previous extracts, where we saw evidence for clinician–patient disalignment in the form of delays and minimal agreement tokens, here the patient's response is comparatively aligning: It is delivered almost contiguously and, with emphasis on the ‘Y’ of ‘yes’, agrees unequivocally that she is overweight (line 14). The clinician discusses risks, as before.

One explanation for the comparative ease with which the patient responds to this method for introducing her possible weight problem is that, seemingly, asking the patient whether she is overweight gives her more agency than simply announcing that she is overweight: With a yes/no question, the patient is treated as having epistemic access to, and primacy over, the assessable, and hence the knowledge and right to respond ‘yes’ or ‘no’. The questioner, by contrast, is positioned as lacking that knowledge and in an epistemically inferior position (Heritage & Raymond, 2012). This is in contrast with an announcement, where the clinician has already decided that the patient is overweight, they position themselves as having epistemic primacy over the assessable, and the assessment is presented to the patient as a *fait accompli*.

However, despite being designed to convey an *unknowing* epistemic stance, conversation analysts have shown that polar (yes‐no) questions like ‘are you overweight?’ are grammatically built to constrain respondents to *confirm* the presuppositions within them (in this case a negative presupposition – they are overweight) (Heritage, 2010; Heritage & Raymond, 2012). Here, the ‘(“yes”‐inviting)’ form of the question ‘are you overweight’ ‘cross‐cuts its negative socio‐medical preference’ (Heritage, 2010). This grammatical constraint is further validated by the fact that (1) assessments about weight are often made on the basis of visual inspection alone (or other objective evidence available to the clinician), and (2) by the clinician's agenda to convey the risks of being overweight (lines 18 onwards). Hence in this context, the question ‘are you overweight?’ appears to be an ‘exam’ or ‘known information’ question for which there is a correct answer (Mehan, 1979; Stokoe & Edwards, 2008).

The patient's response in extract 2b neatly demonstrates how these are also live issues for patients.


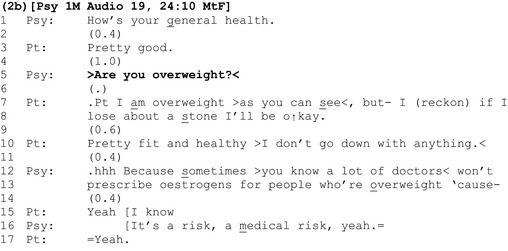


The patient confirms that she is overweight (line 7). However, her agreement stands out because it *repeats* the assessment, ‘I am overweight’ rather than simply stating a type conforming ‘yes’. By doing so, the patient asserts *her* agency and greater epistemic authority over the assessment than that conceded by the clinician in the design of the question (Heritage & Raymond, 2012).

Unlike yes/no responses, ‘repetitive responses’ like these demonstrate resistance to the terms of the question and ‘are associated with sequence expansion’ (Heritage & Raymond, 2012). Here, the patient expands her response by highlighting the ‘exam’ or ‘known answer’ status of the question (‘as you can see’, line 7), before qualifying her agreement, providing her own assessment of the amount of weight that she estimates she needs to lose to ‘be okay’ (lines 7–8).

Instead of agreeing with her assessment, the clinician allows a gap to materialize (line 9). The patient expands her response (line 10). However, once again, the clinician allows a gap to materialize (line 11) before sequentially deleting both of the patient's responses, building his account for raising the topic of weight as incrementally continuous with his earlier question (line 12).

Extract 2c shows the same clinician using a multi‐unit interrogative to introduce the idea that the patient is overweight.


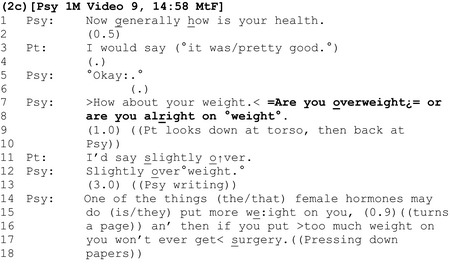


As we have already demonstrated, the question ‘are you overweight’ is grammatically tilted towards (it ‘prefers’) an agreeing response. By contrast, the interrogative shown here – ‘Are you overweight¿= or are you alright on °weight°’ (lines 7–8) offers two candidate answers from which the patient can choose, thereby making it easier for her to state that she is *not* overweight by reducing the grammatical constraint to agree with the first option (Heritage, 2010). In contrast to patients’ relatively contiguous, agreeing responses in extracts 2a and b, here there is a long delay (line 9) before the patient accepts the apparent freedom provided by the question and offers the moderated response that she is ‘slightly over’ (line 11).

Instead of acknowledging the patient's response (e.g., with ‘okay’), the clinician asserts his epistemic authority over the assessment by repeating it, using the full‐term ‘overweight’ (line 12), before discussing the risks associated with excess weight using a threat (lines 16–17).

In extract 2d, the clinician uses an interrogative to topicalize the patient's weight that contains the colloquial term ‘fat’.


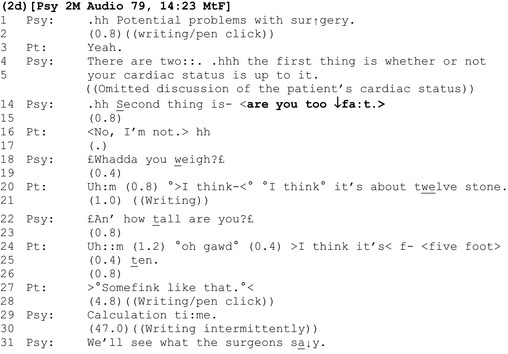


Like ‘are you overweight?’, ‘are you too ↓fa:t’ is designed for an agreeing response (Heritage, 2010). Indeed, we can infer from both the composition and placement of the interrogative that it reflects a presumption by the clinician that the patient is indeed ‘too fat’: Compositionally, unlike ‘are you fat?’ the question ‘are you *too* fat?’ suggests that the matter for consideration is not whether the patient is fat, but whether she is *excessively* fat. In terms of its placement, the possibility of being ‘too fat’ is presented as the second of two ‘potential *problems* with surgery’ (lines 1, 4, and 14, emphasis added).

Perhaps unsurprisingly given the derogatory connotations of ‘fat’, a delay follows this assessment (line 15), and in the only example in the corpus where a patient actively resists the presumption implied by the question, she strongly disagrees that she is fat, asserting her own authority on the matter (‘No, I'm not’, line 16). Rather than accepting this, the clinician defers his response pending further evidence in the form of a BMI calculation (lines 18 onwards).

Although not verbalized by the clinician, our calculation shows a healthy weight. Interestingly, instead of announcing this positive outcome to the patient, the clinician withholds it, deferring the decision regarding whether the patient is overweight to the surgeons (line 31).

The final analytic section contains further examples of clinicians using a BMI calculation to initiate discussion of a possible weight problem.

### Deducing that patients are overweight or obese by calculating their BMI

In extract 3a, weight is introduced via a BMI calculation following some questions about the patient's background.


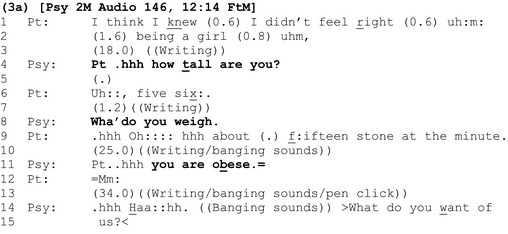


Unlike the standalone announcements in the first analytic section which *initiate* discussion about weight, here the questions ‘how tall are you’ (line 4) and ‘wha’ do you weigh’ (line 8) allow clinician and patient to participate jointly in a step‐by‐step calculation that *results in* an announcement that the patient is ‘obese’ (line 11). The collaboratively arrived at, objectified assessment secures immediate acknowledgement from the patient (line 12), although on this occasion, the clinician does not use the calculation as a platform for further discussion of surgical risks.

Extract 3b shows the same clinician calculating another patient's BMI.


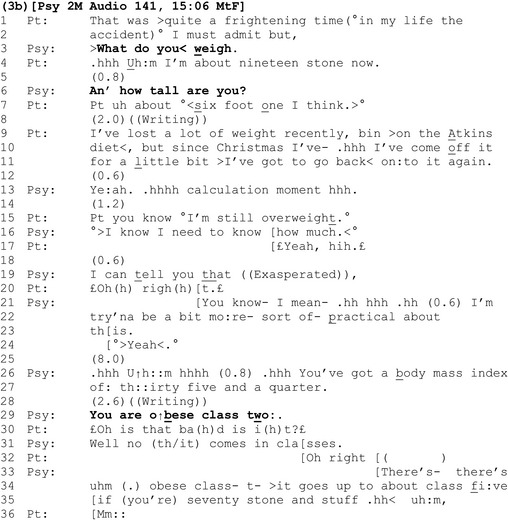


During the calculation, the patient produces an account of recent weight loss (lines 9–11). This can be understood as a defensive move against the upcoming, potentially threatening outcome of the calculation, that she is overweight, and undercuts any presumption by the clinician that she is not already addressing this (cf. extracts 1c and 2a).

Interestingly, here again (see also extract 2b), the patient orients to the upcoming result as already known by the clinician: ‘you know °I'm still overweight.° (line 15). However, here the epistemic tussle over what is already known, who knows it, and who has primacy over that knowledge is made explicitly relevant (lines 15–19). The clinician asserts primacy over the information (lines 16 and 19), justifying this approach by reference to its practicality (lines 21–23). After informing the patient of her BMI result (lines 26–7), the clinician provides the upshot of this calculation by announcing that she is ‘o ↑ bese class two:’ (line 29).

The patient's response to this announcement is different to those in the first analytic section: Although she has anticipated a result that shows she is overweight (line 15), it is prefaced with a change of state ‘oh’ (line 30), showing that she treats this information (that she is obese class two) as new (Heritage, 1998). Her question ‘is that ba(h)d is i(h)t?£’ (line 30) is articulated using ‘smile voice’ and interpolated laughter, indicating that receiving this information may be delicate (Haakana, 2001). It expands the sequence by seeking further information regarding the severity of the diagnosis, which the clinician provides (lines 31ff).

## Discussion

### Summary

This is the first study to provide a detailed examination of actual clinical consultations in order to understand how doctors in a non‐weight‐specific setting initiate discussions about a patient's possible weight problem. We identified three communication practices used by clinicians to introduce the possibility of a weight problem with patients. Here, we discuss which of these practices appear to work well or less well for clinician–patient alignment.

The first practice – *announcing* that patients are overweight – was arguably the least aligning method used by clinicians; it generated significant delays and comparatively minimal responses from patients. We suggested that announcements position the clinician as having epistemic primacy over the assessable, minimize patient's agency to self‐define as overweight (or not), and constrain them to agree with the clinician's negative assessments. For some patients, being told directly that they are overweight via an announcement of this kind may motivate weight loss (Kushner, 2011). However, this practice comes closest to an authoritarian, ‘unilateral’ (clinician determined) model of communication, which is at odds with the collaborative, ‘bilateral’ (shared) approach to doctor–patient communication explicitly advocated by the NHS, NICE, and in the wider literature (Collins, Drew, Watt, & Entwistle, 2005; Coulter & Collins, 2011; Kushner, 2011; National Institute for Health and Care Excellence, 2006; NHS RightCare, 2017; Strategies to Overcome and Prevent Obesity Alliance, 2014; Swift *et al*., 2013).

In extracts 1a and 1c, clinicians masked the interactional turbulence generated by their announcements, building their next turns as incrementally continuous with prior assessments. Interestingly, however, where clinicians addressed this turbulence ‘head‐on’ by demonstrating attentiveness to the delicacy of the assessment (as in extract 1b), a more aligning response, where the patient unequivocally agrees with the assessment, is secured.

The second practice – *asking* patients whether they are overweight – generated comparatively aligning, contiguous, and non‐minimal responses from patients (extracts 2a and 2b). One explanation for this is that polar questions – on the surface – treat patients as having epistemic access to, and primacy over, the assessable. The questioner, by contrast, is placed in an epistemically inferior position (Heritage & Raymond, 2012). However, despite giving patients the apparent choice between yes and no responses, conversation analysts have shown that polar questions are grammatically built to constrain patients to *confirm* the presuppositions within them (e.g., that they are overweight) (Heritage, 2010; Heritage & Raymond, 2012). Hence, we suggested that the question ‘are you overweight’ appears to be an ‘exam’ or ‘known information’ question for which there is a right answer – something that patients themselves occasionally highlight and resist in their responses (e.g., extract 2b). Where the grammatical constraint to agree with the proposition that the patient is overweight is reduced via an additional turn component (extract 2c), the patient moderates the negative assessment by labelling herself ‘slightly’ overweight.

Extract 2d shows that interrogatives can become problematic when clinicians use the potentially offensive assessment ‘too fat’ (Kushner, 2011; Vallis *et al*., 2013). Indeed, this represents one of the only examples in the corpus where a patient explicitly resists the clinician's negative attribution.

In the final analytic section, we explored instances where apparently ‘objective’ BMI calculations were used to *deduce* that the patient is overweight or obese. Unlike the unilateral announcements in the first section which *initiate* discussion about weight by imposing a negative assessment on the patient (extracts 1a‐1c), BMI calculations allow clinicians and patients to participate jointly in a bilateral, step‐by‐step, medicalized calculation that *results in* the announcement that the patient is ‘obese’. Although the literature recommends avoiding terms such as ‘obese’ (Kushner, 2011; Strategies to Overcome and Prevent Obesity Alliance, 2014), the BMI calculation appears to evade the kinds of interactional problems identified earlier because it deflects responsibility for labelling the patient as overweight or obese (or not) onto an objective instrument.

The interactional practices identified here convey to patients that their weight is problematic (with the notable exception of extract 2d). The final practice – calculating patients’ BMI – is in line with recommendations in the literature to medicalize patients’ weight (Scott *et al*., 2004). However, we could not find any evidence that clinicians employed the other recommended strategies to discuss weight highlighted in the introduction: Clinicians did not seek permission from patients to discuss their weight, or ask patients if they were concerned about the effects of their weight on their health or quality of life (Kushner, 2011; Strategies to Overcome and Prevent Obesity Alliance, 2014; Vallis *et al*., 2013).

### Limitations

This study is limited by the fact that recordings were made at one GIC between 2005 and 2006 (Speer & Green, 2008). Therefore, findings may not reflect what occurs in other settings, and current communication practices may differ. Our aim is not to make generalizations about communication from this one setting that will reflect what occurs across multiple health care environments today. Each clinical setting, including the GIC, has its own unique set of interactional demands that influence the ways in which weight is discussed. Rather, we have highlighted the importance of grounding communication skills training and clinical practice empirically, in recordings of actual consultations.

### Conclusions

When actual examples of communication are subject to analytic scrutiny, findings often depart in significant and sometimes surprising ways from ‘textbook’ theory and guidance (Speer, 2013). We do not use these data to criticize clinicians who have generously exposed themselves to analytic scrutiny. Rather, we hope to have highlighted the value to be gained from grounding investigation of weight communication in the close analysis of empirical examples of weight talk from actual consultations.

We have demonstrated that small differences in the wording of turns that initiate discussions about a possible weight problem can have significant consequences for interactional alignment. Three issues in particular may repay further investigation. First, where clinicians make inaccurate *presumptions* that patients are not addressing their weight (e.g., extracts 1c and 2a) or second, assert their *epistemic primacy* to define patients as overweight or obese, this generates problems for the interaction and potentially for the doctor–patient relationship (e.g., the epistemic tussle in extracts 2b and 3b). Third, where clinicians *attend explicitly to the patient perspective*, by vocalizing the potentially negative inferences associated with their own talk (extract 1b), rather than masking, or sequentially deleting patient's responses (extracts 1a, 1c, and 2b), positive interactional consequences – and consequences for the doctor‐patient relationship – may follow.

### Practice implications

Health psychologists and others have identified an ‘urgent need’ for training in communication techniques for weight management that ‘broach sensitive topics without damaging patient relationships’ (Dewhurst *et al*., 2017: 897) and alerting all trainee health professionals to ‘the potential consequences of their language’ (Swift *et al*., 2013: 189). We hope to have demonstrated the potential value of conversation analysis in this endeavour. In particular, clinicians from a range of specialities may benefit from considering the interactional consequences of different practices for introducing weight during the kinds of real‐life discussions of weight considered here, and their advantages and disadvantages. In doing so, they may reach their own judgements regarding what constitutes good practice, and which practices are optimal for facilitating patient‐centred, collaborative discussions of weight in their own clinical settings. As effective communication is central to health outcomes (Street, Gregory, Arora, & Epstein, 2009), this may be of great significance in tackling overweight and obesity. The call to make ‘every contact count’ makes this an ever more pressing task (National Health Service Future Forum, 2011).

## Contribution

Speer gained ESRC funding to support the study, secured access and ethical approvals, and coordinated data collection. McPhillips conducted the data trawl, transcribed the data shown here, and led the writing of the manuscript for her PhD thesis, under the supervision of Speer. Speer and McPhillips jointly analysed the data. Speer edited successive drafts and compiled and revised the final version of the manuscript.

## Conflict of interest

All authors declare no conflict of interest
